# A Case of Reverse McConnell’s Sign Associated With Acute Respiratory Distress Syndrome and Septic Shock

**DOI:** 10.7759/cureus.52166

**Published:** 2024-01-12

**Authors:** Brett Curtis, Albert Ha, Jeffrey Xie, Robert Hyzy, Adam S Helms

**Affiliations:** 1 Internal Medicine, University of Michigan, Ann Arbor, USA; 2 Cardiovascular Medicine, University of Michigan, Ann Arbor, USA; 3 Pulmonary and Critical Care Medicine, University of Michigan, Ann Arbor, USA

**Keywords:** mcconnell’s sign, acute respiratory distress syndrome (ards), shock, acute right heart failure, right ventricular cardiomyopathy

## Abstract

We present a case of reverse McConnell’s sign, a rare echocardiographic finding of right ventricular apical hypokinesis and basal hyperkinesis, in a patient with acute respiratory distress syndrome and septic shock. Although multiple etiologies were hypothesized, providers attributed this cardiomyopathy to increased right heart afterload from hypoxic pulmonary vasoconstriction. Cardiac function normalized as the patient's respiratory failure and sepsis resolved. This study highlights the value of early echocardiography to help guide management in critical illness. In our case, this finding helped initiate diuresis and establish a baseline for monitoring cardiac function as this patient's critical illness resolved.

Literature has most commonly associated reverse McConnell’s sign with massive pulmonary embolism and, more rarely, takotsubo cardiomyopathy. Given the absence of PE, takotsubo, or other identifiable cause, this case suggests that reverse McConnell’s sign may more generally indicate acutely increased right ventricular afterload rather than a specific diagnosis. When reverse McConnell’s sign is detected, treatment should focus on reversible causes of elevated right heart pressure (e.g., volume overload, PE) and increased pulmonary resistance.

## Introduction

Reverse McConnell’s sign refers to an abnormal cardiac wall motion pattern of right ventricular apical hypokinesis and basal hyperkinesis [1]. Like its “regular” counterpart, it has become associated almost exclusively with pulmonary embolism [2]. However, recent data has questioned its specificity, as it can occur in non-thromboembolic conditions like takotsubo cardiomyopathy [3,4]. We present the first known case of reverse McConnell’s sign occurring in association with acute respiratory distress syndrome and septic shock. This patient's course demonstrates the importance of prompt echocardiography in the intensive care unit.

## Case presentation

A 48-year-old female with decompensated alcohol-associated cirrhosis and opiate use disorder presented to the emergency department (ED) after providers noted her to be hypoxic, hypotensive, and tachycardic before an outpatient esophagogastroduodenoscopy. In the week prior, she had noted increased lower extremity edema, worsening shortness of breath, cough, and orthopnea.

**Figure 1:**
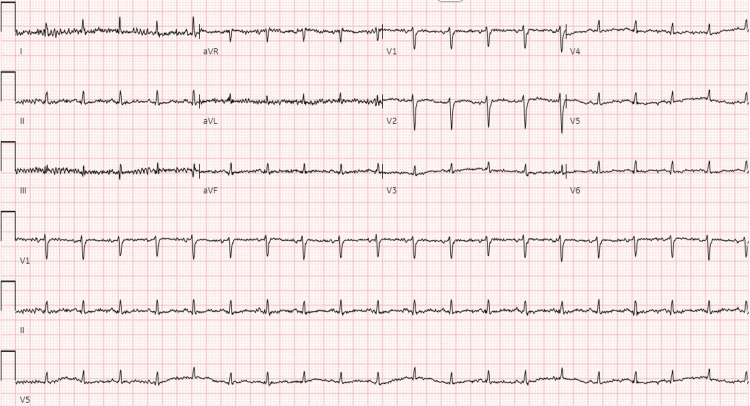
EKG obtained shortly after the presentation to the ED admission. This EKG noted sinus tachycardia with a heart rate of 120 beats per minute and no appreciable ischemic changes.

In the ED, her vitals showed a normal temperature (36.7°C), tachycardia (122 beats/min), tachypnea (21 breaths/min), hypotension (83/53 mmHg), and normal oxygen saturations (SpO_2_ 94%) on room air. She soon required oxygen support via nasal cannula at 2-4 L/min to maintain SpO_2_ 94-96%. Providers discovered 2+ pitting edema to her inguinal region on physical examination. Initial notable lab abnormalities are included below in tabular format with normal reference values for comparison (Table 1). An EKG did not show signs of myocardial ischemia (Figure 1). Chest X-ray showed bilateral opacities, possibly consistent with pulmonary edema, and a right-sided pleural effusion. CT pulmonary embolism protocol (PE) did not detect an acute clot but showed bilateral ground glass opacities and bilateral pleural effusions. CT abdomen/pelvis showed anasarca, ascites, and findings concerning multiorgan hypoperfusion. Finally, a point-of-care ultrasound showed a “D sign” and right ventricular (RV) dilation with grossly normal left ventricular (LV) function, which raised concern for right-sided heart failure from acute or chronic pulmonary hypertension. However, the patient lacked any known prior history of pulmonary hypertension.

**Table 1:** Notable laboratory values at presentation. VBG: venous blood gas; hpf: high-powered field

Lab	Value	Reference range
White blood cell (WBC) count	22.7 k/uL	4-10 k/uL
Neutrophils %	90.6%	36-71%
B-type natriuretic peptide (BNP)	484 pg/mL	100 pg/mL
Procalcitonin	31.9 ng/mL	0-0.25 ng/mL
High-sensitivity troponin, hour 0	40 pg/mL	0-19 pg/mL
High-sensitivity troponin, hour 2	41 pg/mL	0-19 pg/mL
Lactate, VBG	3.1 mmol/L	0.5-2.2 mmol/L
Urinalysis		
Leukocyte esterase	500 U/uL	0 U/uL
Nitrites	Positive	Negative
WBC/hpf	302 WBCs/hpf	0-2 WBCs/hpf
Blood cultures	Gram-negative rods	No organisms

She initially received empiric antibiotics and 1 L of crystalloid for resuscitation to treat presumed septic shock, given her hypotension and laboratory findings concerning UTI. After her respiratory failure continued to worsen and imaging showed signs of right ventricle (RV) failure in the absence of PE, providers administered furosemide with over 2 L of urine output, brief stabilization of SpO_2_ over 93% on 12-15 L non-rebreather mask and blood pressure at 80s-90s/50s-60s mmHg with mean arterial blood pressures consistently over 60 mmHg by non-invasive blood pressure cuff monitoring. Despite treatment, she continued to deteriorate within the first 12 hours of ED presentation, prompting intubation for respiratory failure and initiation of norepinephrine for blood pressure support prior to ICU transfer. At this point, she met the criteria for acute respiratory distress syndrome (ARDS) based on PaO_2_ 119 mmHg and FiO_2_ 50% (P/F 228).

In the ICU, providers continued treatment for presumed septic shock with broad-spectrum antibiotics and initially deferred diuresis given her vasopressor requirement. Blood cultures soon speciated and grew *Escherichia coli*. She self-extubated one day after ICU transfer but was able to maintain adequate oxygen saturations with bilevel positive airway pressure (BiPAP). Transthoracic echocardiography (TTE) performed three days into her ICU stay showed LV ejection fraction (LVEF) of 75%, hyperdynamic LV systolic function based on the aforementioned LVEF, tricuspid annular plane systolic excursion (TAPSE) 24 mm, mild RV enlargement, dilated tricuspid annulus, mild tricuspid regurgitation, right atrial pressure (RAP) 15 mmHg, and moderately decreased RV systolic function with severe mid-apical RV hypokinesis (Videos 1, 2, “reverse McConnell's sign”) [5]. Other measures of RV strain, including pulmonary artery (PA) pressures or measurements of RV:LV size, were not obtained on this TTE.

**Video 1:** Reverse McConnell's sign on subxiphoid TTE. This subxiphoid TTE juxtaposes hyperkinesis of the RV base and hypokinesis of the RV apex while demonstrating normal LV function. TTE: transthoracic echocardiography

**Video 2:** Additional subxiphoid view on TTE. This second subxiphoid TTE better captures this patient's RV dysfunction and normal/hyperdynamic LV function. TTE: transthoracic echocardiography

Her vasopressor requirement stabilized within 12 hours and resolved just over 24 hours after initiation. Providers initiated diuresis and her oxygen requirement improved to nasal cannula. Her antibiotics were narrowed to cefazolin for *E. coli* bacteremia. She also received a right-sided thoracentesis one day after ICU admission that showed transudative fluid based on a ratio of pleural fluid-to-serum protein <0.6. Providers weaned vasopressors off in several days and her oxygen requirement continued to decrease with diuresis and antibiotics. She transferred out of the ICU six days after admission but still required 2-3 L oxygen on nasal cannula at that time.

On the floor, she continued to receive diuresis for her persistent oxygen requirement, which providers attributed to volume overload. A repeat TTE showed LVEF 65% and improvement, but not complete resolution, of RV dysfunction with TAPSE 19, a dilated tricuspid annulus, and minimal tricuspid regurgitation. In consultation, cardiology recommended continued diuresis and attributed her RV failure to acute right heart strain from increased PA pressure in the setting of ARDS. The final TTE, performed shortly before hospital discharge, showed normal biventricular function but detected a small, delayed right-to-left shunt post-valsalva without any evidence of septal defect. This shunt had not been detected on any prior TTEs, which also did not demonstrate the presence of a septal defect. Other measures of RV strain, including TAPSE, were also not reported on this third TTE. Notably, several objective measures of RV strain (e.g., PA pressures) were not reported on any TTE obtained during hospitalization. Invasive hemodynamic measurements via right heart catheterization were not performed because providers did not feel this was indicated for the diagnosis of shock.

Ultimately, she completed a full course of antibiotics for Gram-negative bacteremia. The patient did not require home oxygen on assessment with a six-minute walk test. She was discharged on oral diuretics for ascites and with general cardiology follow-up. She has remained in her baseline state of health with regular healthcare follow-ups since discharge.

## Discussion

To our knowledge, this is the first known case of reverse McConnell’s sign (rMS) reported in association with ARDS and septic shock. Although the cause of this patient’s rMS cannot be definitively determined, the most likely hypothesis attributes this finding primarily to increased PA pressure from hypoxic vasoconstriction and V/Q mismatch in the setting of ARDS, with improvement and normalization of her RV function coinciding with resolution of her respiratory failure. Another possibility indicates that the rMS could represent a stress cardiomyopathy of the RV related to her septic shock. Regardless of the cause, other diagnoses commonly reported with rMS, such as PE or takotsubo cardiomyopathy, were ruled out with imaging and echocardiography. The patient also lacked any prior history of pulmonary hypertension. Although BNP elevation is commonly understood to indicate acute heart failure, levels fell into the "grey zone," a range between 100-500 pg/mL that can be considered inconclusive for heart failure and may more accurately suggest volume overload in the setting of RV dysfunction in this case [6]. Finally, other possible causes, such as LV ischemia or a separate heart failure process, appear unlikely given normal EKG findings, down-trending troponins and lack of chest pain on presentation, serial improvement of her RV function, and maintenance of LV function throughout her illness. Of note, the delayed right-to-left shunt on the last TTE may represent a super-imposed extracardiac shunt from either hepatopulmonary syndrome related to decompensated cirrhosis or portopulmonary syndrome [7].

rMS, named for a pattern of cardiac wall motion of RV apical hypokinesis and basal hyperkinesis that is inverted compared to the usual McConnell’s sign, was first reported in 2005 [1]. Cases are rare in the literature, partially because of difficulty with visualizing the RV on bedside echocardiography [8]. It is most commonly associated with cases of submassive or massive PE and in takotsubo cardiomyopathy, usually in elderly males [1,3,9-11]. While rMS is highly specific for diagnosing massive PE on TTE, it is not always capable of doing so [9].

Early evidence suggests that rMS may be more commonly associated with a higher prevalence of comorbidities, adverse events, and certain biochemical changes, but this data remains mixed [11,12]. Its prognostic and clinical significance otherwise are unknown [13]. Its pathogenesis may be similar to takotsubo cardiomyopathy, which is frequently attributed to a catecholamine surge in the setting of a physical or emotional stressor. However, rMS may also represent a protective mechanism of the RV when subjected to high afterload from abruptly increased PA pressure, which may lead to overstretch-triggered myocardial stunning [14]. Ischemia, caused by increased oxygen demand from increased wall stress, may be the cause of rMS or potentiate pre-existing RV dysfunction [14].

The present case of rMS highlights important aspects of identifying imaging findings that can help guide treatment and early diagnosis in a patient’s course, including additional tests to rule out treatable causes of shock such as PE. Therefore, this case suggests that the management of patients with rMS in critical illness should focus on treatable causes of RV afterload, including but not limited to PE and takotsubo cardiomyopathy. Providers must optimize RV volume status with diuresis or fluid infusion as guided by serial assessment of RV function with echocardiography [15]. When possible, they should also reverse ongoing causes of increased RV afterload. These include pulmonary capillary vasoconstriction, RV outflow resistance (e.g., massive PE), left heart failure, or increased alveolar pressures (e.g., hyperventilation while receiving mechanical ventilation) [16].

## Conclusions

We report the first known instance, to our knowledge, of rMS associated with septic shock and ARDS. Given the absence of venous thromboembolism, this case suggests that rMS should not be viewed as highly specific for PE. Providers should consider it more broadly as a sign of RV strain to assist in managing undifferentiated shock. Detection of rMS should prompt a search for precipitants of heart failure or RV strain, including but not limited to PE. Immediate treatment should focus on treating the underlying cause and optimizing the patient’s volume status as guided by bedside ultrasound. After recovery, appropriate cardiology follow-up remains essential.
